# Prevalence and related factors of anemia in HAART-naive HIV positive patients at Gondar University Hospital, Northwest Ethiopia

**DOI:** 10.1186/2052-1839-13-8

**Published:** 2013-08-09

**Authors:** Getachew Ferede, Yitayih Wondimeneh

**Affiliations:** 1 School of Biomedical and Laboratory Sciences, College of Medicine and Health Sciences, University of Gondar, P.O. Box 196, Gondar, Ethiopia

**Keywords:** Anaemia, HIV, HAART-naïve, CD4 count

## Abstract

**Background:**

Anaemia is a common complication of infection with the human immunodeficiency virus (HIV) and may have various causes. The aim of this study was to determine the prevalence and related factors of anemia in HAART-naive HIV positive Patients.

**Methods:**

A retrospective study was conducted on HAART naive HIV positive patients at the Gondar University Hospital between September 2011 and August 2012. Socio-demographic and immunohematological (hemoglobin and CD4+ T cells) data were collected carefully from the existing ART logbook and patient follow up cards. Anaemia was defined according to the WHO criteria.

**Results:**

The overall prevalence of anaemia was 138 (35%). Female HAART naive HIV positive patients had significantly (P < 0.05) higher prevalence of anaemia than males (62% Vs 38%). The prevalence of anaemia at different CD4 level was; 6 (4%) with CD4 count greater than 500 cells/μL, 18 (13%) with a CD4 count of 350–500 cells/μL, 37 (27%) with a CD4 count of 200–349 cells/μL, 44 (32%) with a CD4 count of 100–199 cells/μL, 14 (10%) with a CD4 count of 50–99 and 19 (14%) with CD4 count of less than 50 cells/μL.

**Conclusions:**

Our findings showed that one-third of HAART naïve HIV positive patients were anaemic and the increase in prevalence of anaemia with decreased CD4 cell count was statistically significant. Therefore, early diagnosis and treatment of anaemia in these patients are essential.

## Background

Human immunodeficiency virus (HIV) infection is frequently associated with hematologic abnormalities such as anaemia. This association may have many causes [1,2]. A systematic review of these abnormalities has shown that the prevalence of anaemia in HIV positive patients ranges from 20-80% and is associated with faster disease progression and mortality [3]. This makes it more common than thrombocytopenia or leucopenia in patients with AIDS [4]. In established HIV infection, lower haemoglobin levels have been shown to correlate with decreasing CD4+ cell counts [5] and many studies have found an association between anaemia during established infection and a faster progression to AIDS and death [6,7]. Therefore, interventions to prevent anaemia may lead to improved health and survival potential of HIV-infected persons [8].

Anaemia has been reported to influence the natural history of HIV disease by increasing the rate of disease progression and mortality in both developed and developing countries [1]. Mocroft *et al.* have demonstrated that severe anaemia is linked with faster rate of HIV disease progression. They have also shown that anaemia is a strong predictor of death [9]. The consequences of untreated anemia may lead to multisystem disabling symptoms and fatigue, exhaustion, increased risk of HIV dementia, poor quality of life and possibly even exacerbates poverty in communities with a high HIV prevalence [10]. On the other hand, survival time in HIV-infected persons may be enhanced after recovery from anemia [11].

Several observational studies have also reported a higher mortality in HIV infected patients from low haemoglobin levels even after adjusting for CD4 cell count and viral load [3,7]. The etiology of anaemia in HIV infection is multifactorial and typically the anaemia may result from low production of red blood cells, increased RBC destruction, or ineffective RBC production [12] and frequently the laboratory features are compatible with anaemia of chronic disease with a low reticulocyte count, normocytic and normochromic red blood cells with normal iron stores and cytokine mediated poor erythropoietin response [13,14].

Other mechanisms for HIV-associated anemia, although uncommon, include vitamin B_12_ deficiency and the autoimmune destruction of erythrocytes [15]. Direct infection of marrow precursor cells [16] has been hypothesized, but not proven. However, studies from developed countries suggest that the use of highly active antiretroviral therapy (HAART) is associated with an increase in haemoglobin concentrations and a decrease in the prevalence of anaemia. Amelioration of HIV-related anaemia with HAART has several benefits including improvements in functional status, energy levels and fatigue and overall improvement in quality of life [16].

However, sufficient treatment of anaemia is not always considered in developing countries. This is because most notice is given to HIV infection and the frequent complications such as opportunistic infections [17]. Moreover, there is wide variation in the prevalence of anaemia among HIV/AIDS patients in different studies all over the world and paucity information on the prevalence of anaemia among HIV patients in Ethiopia. This study therefore seeks to evaluate the prevalence of anaemia and the related factors among HAART-naïve HIV positive patients at different age groups, gender and CD4 levels.

## Methods

A retrospective study of HAART naive HIV positive patients was conducted at Gondar University Hospital, Northwest Ethiopia, between September 2011 and August 2012. Our study participants were adult (18 years or above) HAART naive HIV positive individuals. HIV positive patients who were on antiretroviral therapy and pregnant women were excluded. Pre-ART HIV patients who were on medication such as antibiotics, vitamin supplements and tuberculosis treatments were also excluded from the study.

Participant’s socio-demographic variables, history of prophylaxis taken and hematological (hemoglobin) and immunological (CD4+ T cells) values were carefully extracted from ART log book and patients follow up cards. Absolute counts of CD4 lymphocytes were assayed using the BD FACSCOUNT system (Becton Dickenson and Company, California, USA). Haematological parameters such as haemoglobin (Hb) were determined using the automated blood analyzer Cell-Dyn 1800 (Abott Laboratories Diagnostics Division, USA).

Anaemia was defined according to the WHO criteria [18]. For males, anaemia was defined as haemoglobin concentration (Hb) less than 13 g/dl, while for females; the value is less than 12 g/dl. Mild anaemia was defined as a haemoglobin level of 8-13 g/dl for men and 8-12 g/dl for women. Severe anaemia was defined as a haemoglobin level of less than 8 g/dl for both males and females.

Data were entered and analyzed by using SPSS version 16. Descriptive statistics (minimum, maximum, mean and standard deviation) were determined for continuous variables in the course of analysis. Proportions and percentages were calculated for categorical variables. For all statistical comparisons of the groups, the Pearson Chi-square test was used and P-value < 0.05 was considered as statistically significant.

Ethical clearance was obtained from the Institutional Ethical Review Board of University of Gondar. Permission for the conduct of the study was also obtained from the University Hospital.

## Results

A total of 420 HAART naive HIV positive patients’ chart were reviewed but 20 of them were excluded from the study due to co- infection, opportunistic infection, pregnancy and those who were on medication (antibiotics and vitamin supplements). The overall prevalence of anaemia was 138 (35%) and majority of patients had mild to moderate anemia. Mild to moderate anemia and severe anemia occurred in 128 (32%) and 10 (3%) patients, respectively. Female HAART naive HIV positive patients had significantly higher prevalence of anaemia than males (62% Vs 38%) (P<0.05) (Table 1, 2 and 3).

**Table 1:** Socio-demographic characteristics of HAART naive HIV positive patients at Gondar University Hospital, Northwest Ethiopia, 2012

**Variables**	**Frequency**	**Percentage (%)**
**Age (years)**		
18-29	128	32
30-39	168	41.8
40-49	78	19.5
50 & above	27	6.8
**Sex**		
Male	122	30.5
Female	278	69.5
**Residence**		
Urban	335	83.8
Rural	65	16.2
**Religion**		
Christian	368	91.8
Muslim	31	7.7
Others	2	0.5
**Marital status**		
Married	200	50
Single	75	18.8
Divorced	82	20.5
Widowed	43	10.8

**Table 2:** Prevalence of anaemia among HAART naive HIV positive patients at Gondar University Hospital, Northwest Ethiopia, 2012

**Degree of anemia**	**Frequency**	**Percentage (%)**
Normal	262	65
Mild to moderate anemia	128	32
Severe anemia	10	3
Total	400	100

**Table 3:** Prevalence of anaemia by gender among HAART naive HIV positive patients at Gondar University Hospital, Northwest Ethiopia, 2012

**Sex**	**Frequency of anaemia**	**Percentage (%)**	**P – value**
Male	53	38	0.013
Female	85	62	

Hemoglobin levels of the study participants were between 4.7 and 18 g/dL with the mean of 12.6 ± 2.0 g/dl. The minimum CD4 count was 6 cells/μL, and the maximum was 1193 cells/μL. The mean CD4 count was 288±190.2 cells/μL. In this study, majority of the anaemic cases 62 (45%) were observed in the age group of 30–39 years. However, the difference was not statistically significant (Table 4).

**Table 4:** Relationship between age groups and anaemia among HAART naive HIV positive patients at Gondar University Hospital, Northwest Ethiopia, 2012

**Age group**	**Frequency of anaemia**	**Percentage (%)**	**P – value**
18-29	38	28	0.117
30-39	62	45	
40-49	24	17	
50 & above	14	10	

In this study, the prevalence of anaemia was showed statistical significance (P<0.05) with CD4 T-cell count. We found that 6 (4%) with CD4 count greater than 500 cells/μL, 18 (13%) with a CD4 count of 350–500 cells/μL, 37(27%) with a CD4 count of 200–349 cells/μL, 44 (32%) with a CD4 count of 100–199 cells/μL, 14 (10%) with a CD4 count of 50–99 and 19 (14%), with CD4 count of less than 50 cells/μL (Table 5).

**Table 5:** Relationship between varying degrees of CD4 count and anaemia among HAART naive HIV positive patients at Gondar University Hospital, Northwest Ethiopia, 2012

**CD4 count**	**Frequency of anaemia**	**Percentage (%)**	**P – value**
>500	6	4	0.001
350-500	18	13	
200-349	37	27	
100-199	44	32	
50-99	14	10	
<50	19	14	

## Discussion

Anaemia is frequently encountered aberration in HIV patients [19] which may be clinically important. Multifactorial causes of anaemia may complicate its original cause and/or its suitable treatment [20]. The prevalence of anaemia in this study was 138 out of 400(35%) which is in agreement with study of Mildvan *et al.,*[21] and Levine *et al.,*[22]. However, low prevalence was reported by Semba *et al*., who concluded that anaemia was in 28.1% of HIV patients [23]. In addition Mata-Marin *et al.,*[24] and Akinsegun *et al.,*[25] reported 20% and 24.2% prevalence of anemia among HAART naive HIV patients respectively, which was lower than our study**.**

In this study, females had significantly higher prevalence of anaemia than male patients 85 (62%) Vs 53 (38%) (p<0.05), which is in agreement with findings of Owiredu *et al.,*[26] and Nadler *et al.,*[27]. Moreover, mostly female gender has been reported as a risk factor for anaemia among HIV patients [27]. However, the finding in this study was in contrast to the findings of a study conducted in Nigeria [28].

In this study, as the immunity of a patient decreases, anaemia was more prevalent in patients rather than in HIV positive patients who have relatively high CD4 count. For example, the prevalence of anaemia was highest among patients who had a CD4 lymphocyte count of 100–199 cells/μL and lowest among patients with a CD4 count > 500 cells/μL,. The increase in prevalence of anaemia with decreased CD4 cell count was statistically significant (P < 0.05). Mostly, prevalence of anemia is higher among the study participants who have low CD4 count. This finding is consistent with the results of Levine *et al.,*[22] and Volberding *et al.*, [29] who reported that more severe levels of anemia are found among HIV positive patients presenting with low CD4 counts.

The pathogenesis of HIV- related anemia is indistinct and is possible to be multifactorial in nature [1,30,31] Possible causes are bleeding (gastrointestinal malignancy/severe infection), insufficient dietary intake (vitamins such as cobalamin and folate, iron, and general malnutrition), haemolytic anaemia (i.e. malignancies, infections, splenomegaly, immune dysfunction), changes in erythropoietin synthesis and/or bone marrow suppression. Suppression of the bone marrow in HIV-infected patients may be initiated in several ways. These include an action of HIV itself (infection of the progenitor of the red blood cell synthesis in late-stage disease), the use of antiretroviral drugs (primarily zidovudine), direct pathogenic involvement of the bone marrow (malignant lymphoma, atypical mycobacteriosis), prophylactic or therapeutic treatment against opportunistic diseases or malignancies which may suppress red blood cell production [23,32], however since the HIV positive patients in this study were HAART-naive and also devoid of coinfection with opportunistic diseases, the anaemia could be associated with the low CD4 count, which concur with other studies [33,34].

To the best of our knowledge, this is the first study in Ethiopia to determine prevalence of anaemia in HIV HAART-naive infected patients. However, this study had limitations such as the inability to estimate erythropoietin levels and viral load. In addition, this study did not include HIV infected patients who were on HAART.

Farther longitudinal studies should be conducted to develop interventions aimed at reducing the prevalence of anaemia in HIV infected patients. Moreover, studies on serum iron, ferritin, and total iron binding capacity, folate, cobalamin level and bone marrow could give more information on the aetiology of anaemia in HIV infected individuals. These limitations notwithstanding, the results of this study area true reflection of the circumstances of the study participants and thus this study could serve as a reference for additional recommendations to improve care of HIV infected persons and a step for further studies on the pathophysiology of HIV associated anaemia in Ethiopia.

## Conclusions

In conclusion, our findings showed that one-third of HAART naive HIV positive patients in Northwest Ethiopia are anaemic and the increase in prevalence of anemia with decreased CD4 cell count was statistically significant. Sex variation in the prevalence of anaemia was observed with significant higher prevalence of anaemia observed among females. Therefore, early diagnosis and treatment of anaemia is essential in these patients.

## Competing interests

The authors declared no conflicts of interest with respect to the authorship and/or publication of this article.

## Authors’ contributions

GF: Participated in conception and design of the study, data collection, analysis and interpretations of the findings, drafting the manuscript and write up. YW: Participated in conception and design of the study, data collection, analysis and interpretations of the findings, reviewed the manuscript. Both authors read and approved the final manuscript.

## Pre-publication history

The pre-publication history for this paper can be accessed here:

http://www.biomedcentral.com/2052-1839/13/8/prepub
